# Evaluating the morphological features of the lateral pterygoid insertion into the medial surface of the condylar process

**DOI:** 10.1002/cre2.353

**Published:** 2020-11-17

**Authors:** Sasin Sritara, Masahiro Tsutsumi, Keiko Fukino, Yoshiro Matsumoto, Takashi Ono, Keiichi Akita

**Affiliations:** ^1^ Department of Orthodontic Science, Graduate School of Medical and Dental Sciences Tokyo Medical and Dental University Tokyo Japan; ^2^ Department of Clinical Anatomy, Graduate School of Medical and Dental Sciences Tokyo Medical and Dental University Tokyo Japan

**Keywords:** jaw movement, mandibular condyle, masticatory muscles, pterygoid muscles, temporomandibular joint, x‐ray microtomography

## Abstract

**Objective:**

The lateral pterygoid is vital in coordinating multidimensional jaw movements. Since a vector in three‐dimensional (3D) space is defined by two 3D points, the role of the lateral pterygoid in 3D jaw movements is defined by its origin and insertion. Reportedly, the lateral pterygoid is inserted into not only the pterygoid fovea but also into the medial surface of the condylar process. The objective was to investigate the morphological features of the region of the lateral pterygoid that inserted into the medial surface of the condylar process.

**Material and Methods:**

Ten head halves of seven cadaveric donors were analyzed. In all specimens, the insertion area on the medial surface of the condylar process was measured based on microcomputed tomography images. Muscle fibers were separated into ≥50 small bundles, and positional relationships between the origins and insertions were investigated.

**Results:**

Overall, 28.8 ± 5.0% of the insertion area of the lateral pterygoid was situated on the medial surface of the condylar process. Moreover, muscle fiber arrangement revealed that origins of the muscle bundles inserting into the medial surface in seven specimens posteriorly occupied the lateral plate of the pterygoid process longitudinally, whereas those in three specimens mainly occupied the posteroinferior portion.

**Conclusions:**

Muscle bundle inserting on the medial surface of the condylar process had a broad insertion area and a distinct origin on the posterior region of the lateral plate of the pterygoid process. This muscle bundle could act as one of the significant subunits within the lateral pterygoid. Therefore, anatomical coordination mechanisms underlying jaw movement cannot be elucidated without considering this subunit.

## 1 INTRODUCTION

Jaw movements consist of protrusion and retrusion, lateral excursion, and jaw‐opening and closing. These components are three‐dimensionally coordinated by the anatomical structures within the so‐called Posselt's envelope of motion (Posselt, 1957). The lateral pterygoid, which is the one of the masticatory muscles, plays an essential role in predominant components, namely protrusion, lateral excursion, and jaw‐opening (Bhutada et al., 2007; Phanachet et al., 2002; van Eijden et al., 1995, 1997; Yamaguchi et al., 2006). Based on these functional research studies, the anatomical structure of the lateral pterygoid may be adaptive to coordinate the multidimensional jaw movements.

Since a vector in three‐dimensional (3D) space is defined by two 3D points, the role of the lateral pterygoid in 3D jaw movements is defined by its origin and insertion. In general, the two heads of the lateral pterygoid, upper and lower heads, originate from the inferior surface of the greater wing and the lateral plate of the pterygoid process, and converge and insert into the articular disc of the temporomandibular joint and pterygoid fovea of the condylar process (Eisler, 1912; Evans, 2016; Henle, 1855). Recent anatomical studies have reported that the lateral pterygoid not only inserted into the pterygoid fovea but also into the medial surface of the condylar process, which faced a three‐dimensionally different plane from the pterygoid fovea (Akita et al., 2019; Matsunaga et al., 2009; Sakaguchi‐Kuma et al., 2016; Usui et al., 2008). However, the morphological characteristics of the region of the lateral pterygoid that inserts into the medial surface of the condylar process, particularly the size of the insertion area and its muscular fiber arrangement, remain unclear. Its precise characteristics may provide a better anatomical background of the contribution of the lateral pterygoid to the coordination of jaw movement.

In the present study, we investigated the morphological features of the region of the lateral pterygoid which inserted into the medial surface of the condylar process, focusing on the size of the insertion area and the muscular fiber arrangement. We hypothesized that this part of the lateral pterygoid had a broad insertion area on the medial surface of the condylar process and had a distinct origin from the remaining region.

## 2 MATERIALS AND METHODS

### 2.1 Cadaveric specimen preparation

Head halves (14) of 10 Japanese cadavers (six males, four females; mean age at death 77.9 years), donated to the Department of Anatomy, were used in this study. The study design was approved by our institution's Ethics Committee.

**FIGURE 1:**
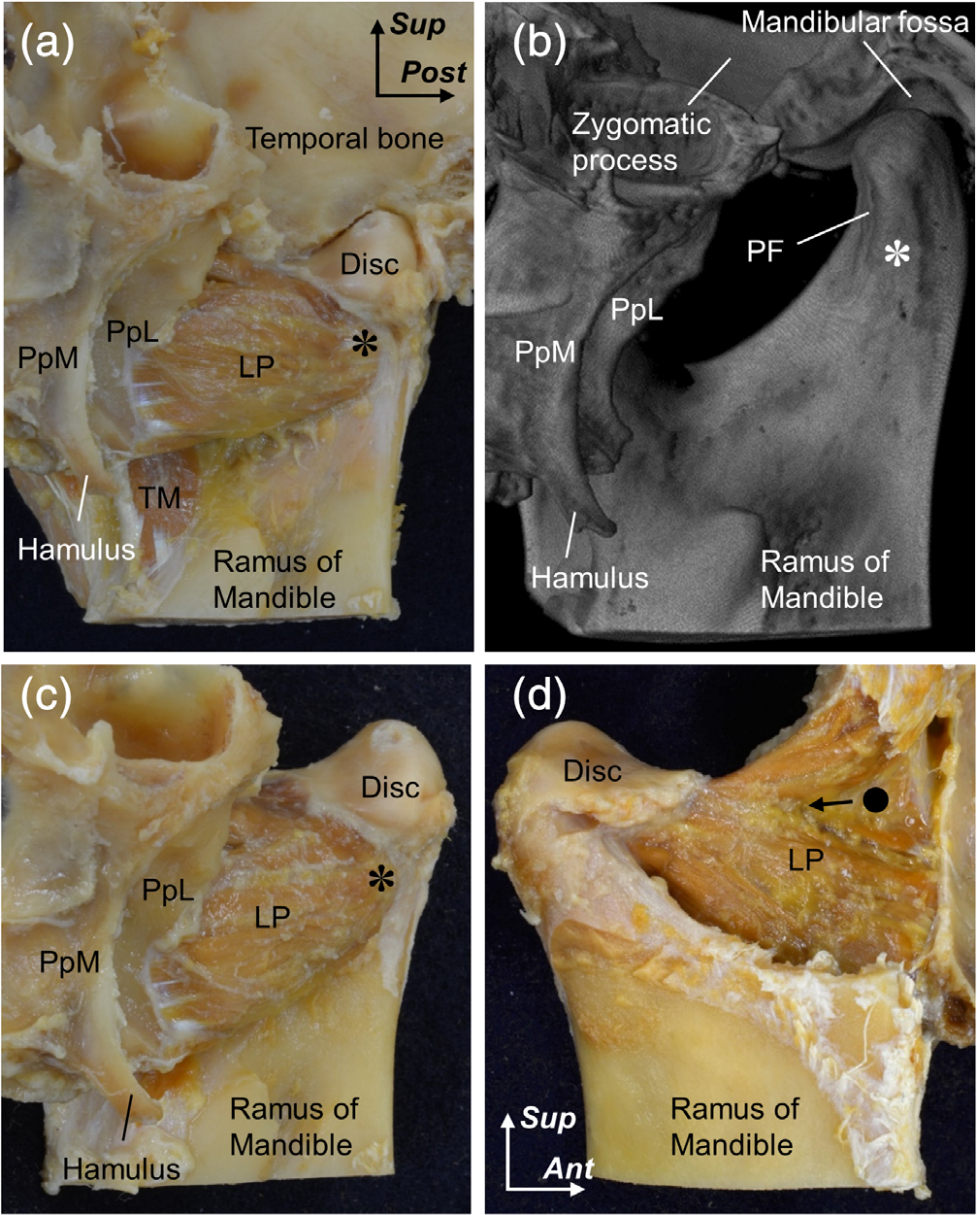
Method of preparation for the detailed mapping of the muscle fiber arrangement. Medial (a–c) and lateral (d) aspects of the right ramus of the mandible. After exposing the medial surface of the lateral pterygoid (LP) and identifying its attachment sites (a), the bony configurations of the attachment sites are examined using micro‐CT (b). The asterisk indicates the medial surface of the condylar process corresponding to the region of LP insertion. After taking micro‐CT, the bony elements (except the attachment sites) are removed to facilitate observation of the muscle fiber arrangement of the medial and lateral aspects of the LPs (c and d). Circle, muscular groove between the upper and lower heads of the LPs; Disc, articular disc of the temporomandibular joint; Hamulus, pterygoid hamulus; PF, pterygoid fovea of the condylar process; PpL, lateral plate of the pterygoid process; PpM, medial plate of the pterygoid process; TM, temporal muscle. *Ant*, anterior; *Post*, posterior; and *Sup*, superior

All cadaver specimens were fixed in 8% formalin and preserved in 30% ethanol. The bony elements were removed from inside the cranium to expose the medial surface of the lateral pterygoid, and its attachment sites were identified (Figure 1(a)). The bony configurations of the attachment sites were examined using microcomputed tomography (micro‐CT) (inspeXio SMX‐100CT Micro Focus X‐Ray CT System; Shimadzu, Kyoto, Japan) with a 200 μm resolution.

Three‐dimensional images were reconstructed using ImageJ software (version 1.52; National Institutes of Health, Bethesda, MD) (Figure 1(b)). After the micro‐CT assessment, the bony elements, with the exception of the attachment sites, were again removed to facilitate observation of the muscle fiber arrangement of the lateral pterygoid (Figure 1(c) and (d)). Additionally, the micro‐CT images of the attachment sites of four specimens revealed severe calcification on the condylar processes. Therefore, only 10 specimens (five right and five left) from seven cadavers (five males and two females; mean age at death 75.1 years) were used for the analyses.

### 2.2 Micro‐CT analysis: Measurement of the insertion area of the lateral pterygoid

**FIGURE 2:**
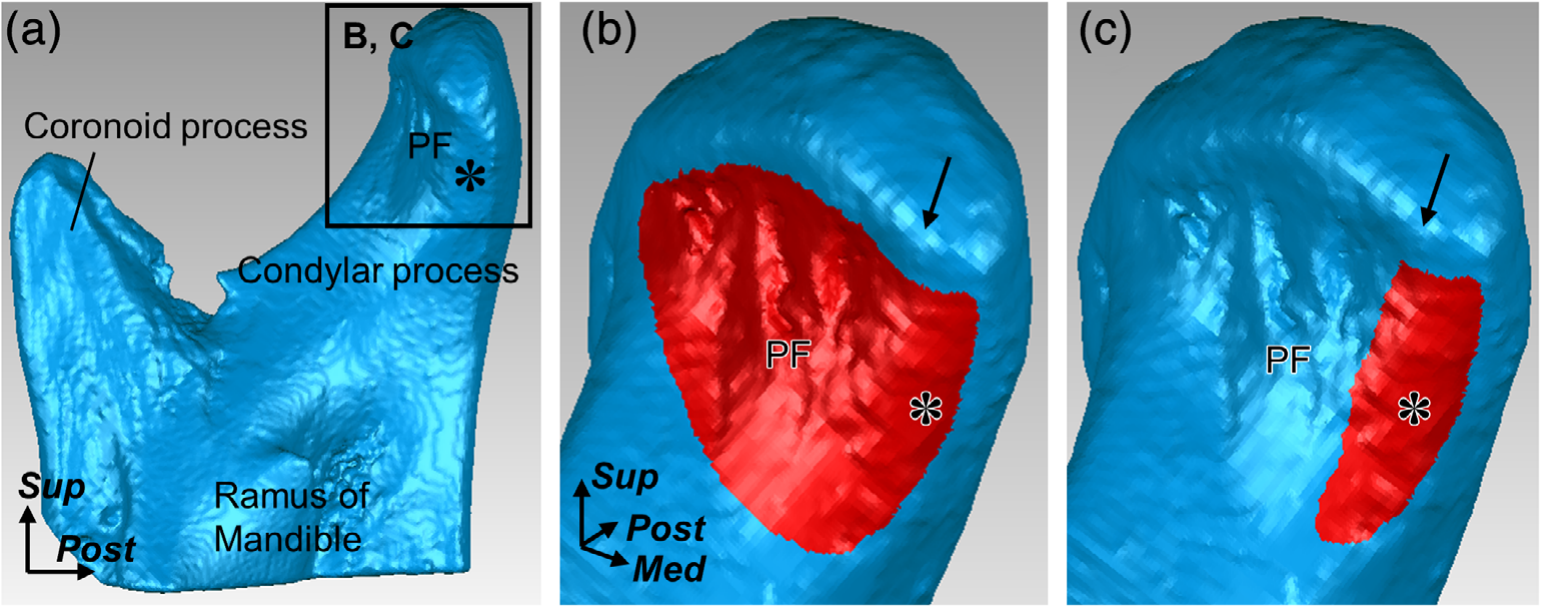
Measurement of the insertion area of the lateral pterygoid on the condylar process. Three‐dimensional surface models of the right ramus of the mandible are created based on the micro‐CT data. (a) Medial aspect of the ramus of the mandible. (b and c) Anteromedial aspect of the boxed region in (a). Red regions indicate the measurement areas of the whole insertion of the lateral pterygoid (b) and the part of its insertion on the medial surface of the condylar process (c). Arrows indicate the inflection points on the mandibular head between the pterygoid fovea (PF) and medial surface of the condylar process (indicated by asterisk). *Med*, medial; *Post*, posterior, and *Sup*, superior

In all 10 specimens, the insertion areas of the lateral pterygoid were measured based on the micro‐CT images. The obtained images were segmented, and three‐dimensional surface models of the ramus of the mandible were created using the OsiriX (Pixmeo, Benex, Switzerland). The three‐dimensional surface models were transferred to the Geomagic Wrap software (3D Systems, Rock Hill, SC) (Figure 2(a)), and the entire insertion (Figure 2(b)), the insertion on the pterygoid fovea, and the medial surface (Figure 2(c)) of the condylar process were measured three‐dimensionally. Data were reported as means ± standard deviation.

### 2.3 Macroscopic analysis: Muscle fiber arrangement of the lateral pterygoid

**FIGURE 3:**
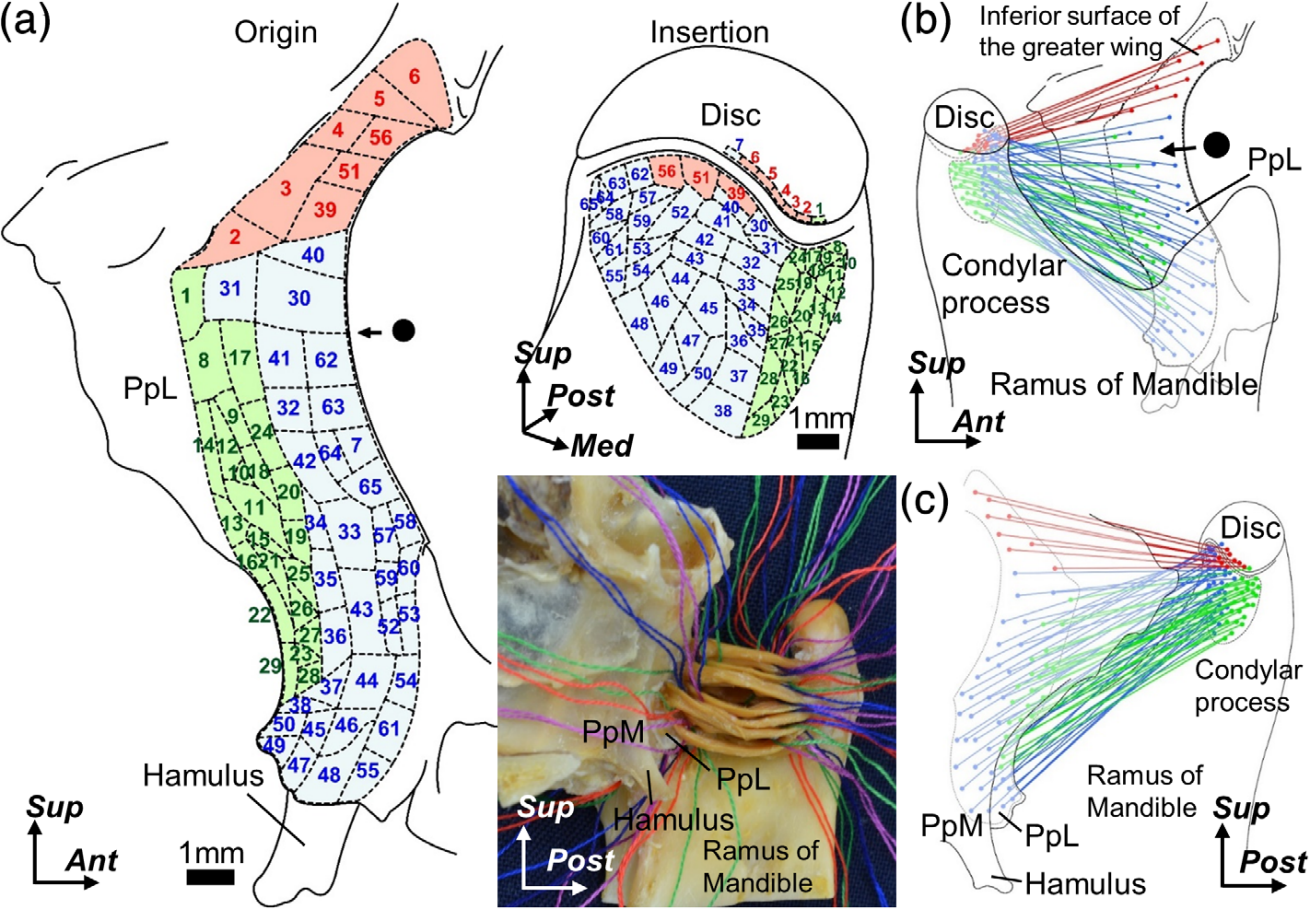
Muscle fiber arrangement of the lateral pterygoid. (a) (Lower right panel) Medial aspect of the right ramus of the mandible. Muscle fibers of the lateral pterygoid are divided into 50 or more small bundles of 1 mm in diameter using strings while maintaining preservation of the attachment sites. (Left and upper right panels) Attachment sites of each small bundles are recorded in the schematic illustrations based on the micro‐CT images of the origin (left: lateral aspect of the sphenoid) and insertion (upper right: anteromedial aspect of the condylar process). Corresponding numbers for each small bundle at the origin and insertion are labeled. Red regions indicate the attachment sites of the small bundles originating from the inferior surface of the greater wing. Blue regions indicate the attachment sites of the small bundles which originated from the lateral plate of the pterygoid process (PpL) and inserted into the pterygoid fovea of the condylar process. Green regions indicate the attachment sites of the small bundles which originated from the PpL and inserted into the medial surface of the condylar process. B and C: Positional relationships between the origin and insertion of the small bundles on the lateral (b) and medial (c) aspects of the lateral pterygoid. Circle, muscular groove between the upper and inferior heads of the lateral pterygoid; Disc, articular disc of the temporomandibular joint; Hamulus, pterygoid hamulus; PpM, medial plate of the pterygoid process. *Ant*, anterior; *Med*, medial; *Post*, posterior, and *Sup*, superior

Muscle fiber arrangements of the lateral pterygoid were also analyzed in all 10 specimens. The muscle fibers of the lateral pterygoid were separated into ≥50 small bundles (1 mm in diameter) while preserving the attachment sites (Figure 3(a)). These preparations were performed by laying a string alongside each bundle course. The number of bundles, the defined bundle diameter, and preparations followed those reported in previous anatomical studies (Hara et al., 2009; Hatsushika et al., 2013; Usui et al., 2008). After preparation, the attachments of both the origin and insertion of each small bundle were recorded. We then investigated the positional relationship between the origin and insertion of each small bundle.

## 3 RESULTS

### 3.1 Measurement of the insertion area of the lateral pterygoid

The pterygoid fovea of the condylar process showed a bony depression corresponding to the insertion area (Figure 2(a) and (b)), whereas the medial surface of the condylar process showed a bony impression or ridge corresponding to the insertion area (Figure 2(a) and (c)). The entire insertion area was 2.0 ± 0.4 cm^2^ and the insertion areas on the pterygoid fovea and medial surface of the condylar process were 1.4 ± 0.3 and 0.6 ± 0.2 cm^2^, respectively. The insertion areas on the medial surface of the condylar process occupied 28.8 ± 5.0% of the total insertion areas.

### 3.2 Muscle fiber arrangement of the lateral pterygoid

Based on the origin on the sphenoid, the lateral pterygoid was divided into the parts originating from the inferior surface of the greater wing and those from the lateral plate of the pterygoid process (Figure 3(b) and (c)). The region originating from the inferior surface of the greater wing inserted into the anterior portion of the articular disc and the most superior portion of the pterygoid fovea. The region originating from the lateral plate of the pterygoid process was subdivided into two parts, which inserted into the pterygoid fovea and the medial surface of the condylar process.

**FIGURE 4:**
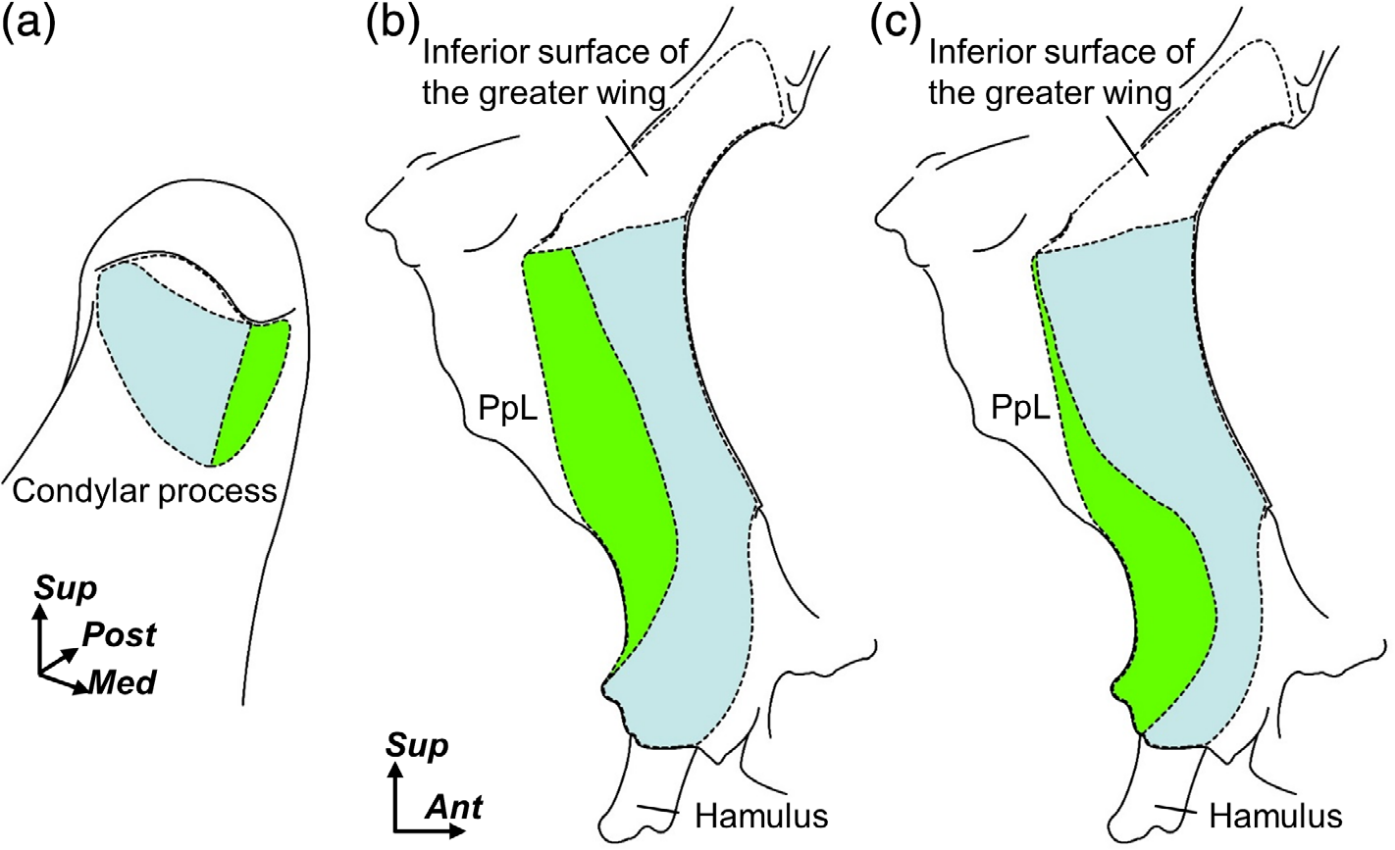
Variation of the origin of the muscle bundles inserting into the medial surface of the condylar process. Schematic illustrations of the anteromedial aspect of the right condylar process (a) and lateral aspects of the right sphenoids (B and C). Blue and green regions indicate the pterygoid fovea and the medial surface of the condylar process. Blue regions indicate the attachment sites of the muscle bundles which originated from the lateral plate of the pterygoid process (PpL) and inserted into the pterygoid fovea of the condylar process. Green regions indicate the attachment sites of the muscle bundles which originated from the PpL and inserted into the medial surface of the condylar process. Two patterns of these origins, posterior (b) and posteroinferior (c), are shown. Hamulus, pterygoid hamulus. *Ant*, anterior; *Med*, medial; *Post*, posterior; and *Sup*, superior

The two patterns of the origin of the region which inserted into the medial surface of the condylar process were observed: posterior (Figure 4(a) and (b)) and posteroinferior (Figure 4(a) and (c)). In the posterior types, the origin occupied the posterior portion of the lateral plate of the pterygoid process longitudinally in seven of the 10 specimens. In the posteroinferior types, the origin mainly occupied the posteroinferior portion of the lateral plate of the pterygoid process in three of the 10 specimens. Both types of origins occupied the posterior half of the lateral plate of the pterygoid process, and these superior portions extended to the inferior margin of the inferior surface of the greater wing.

## 4 DISCUSSION

The micro‐CT analysis in the present study revealed that the insertion area of the lateral pterygoid on the medial surface of the condylar process occupied approximately one‐third of the total insertion. Furthermore, the part of the lateral pterygoid inserting into the medial surface of the condylar process originated from the posterior half of the lateral plate of the pterygoid process. This origin varied according to the individuals, although it was distinguishable from the origin of the remaining region.

Data on the dimensions of the insertion area of the lateral pterygoid is limited in the literature. Recently, She et al. (2018) reported that the total insertion area of the lateral pterygoid was 1.4 cm^2^ based on the three‐dimensional cone‐beam CT images. However, the insertion areas evaluated in the present study were 2.0 cm^2^ and were therefore larger than those of the aforementioned study. Since the insertion area on the pterygoid fovea in the present study was 1.4 cm^2^ and almost identical with the study of She et al. (2018), the discrepancy might be explained by whether or not there was specific focus on the insertion area on the medial surface of the condylar process. On the basis of occupying one‐third of the total insertion area, we concluded that the lateral pterygoid had a broad insertion area on the medial surface of the condylar process which should not be ignored.

Regarding the muscle fiber arrangement of the lateral pterygoid, Usui et al. (2008) reported that the muscle bundles, which originated from the posterior half of the lateral plate of the pterygoid process, mainly inserted into the medial surface of the condylar process. However, this muscle fiber arrangement was analyzed based on the subdivision of the origins on the sphenoid. Therefore, Usui et al. (2008) did not show the precise origin of the muscle bundle inserting into the medial surface of the condylar process. The present study focused on the muscle bundle that inserted into the medial surface of the condylar process and revealed that in some specimens, the origin occupied the posterior portion of the lateral plate of the pterygoid longitudinally, and in the others, it mainly occupied the posteroinferior portion. Specifically, the muscle bundle inserting into the medial surface of the condylar process can be regarded as having a distinct origin from the remaining region, regardless of the variations among individuals.

**FIGURE 5:**
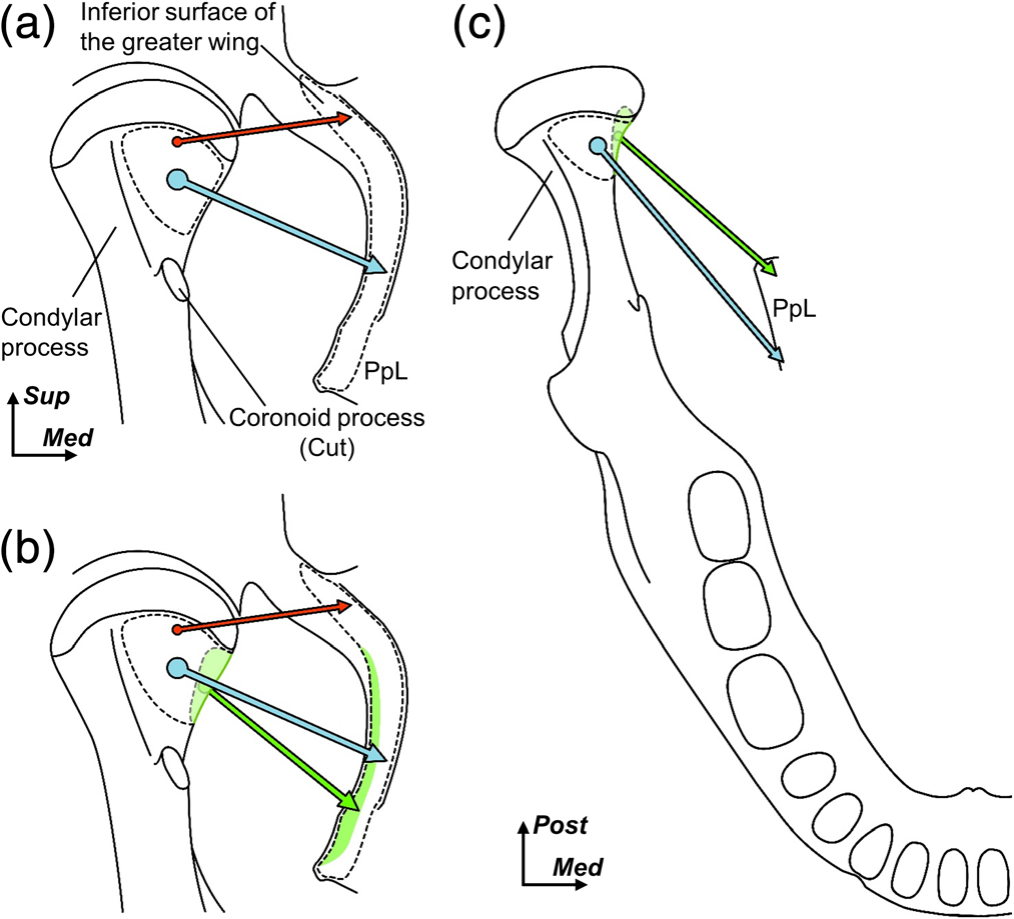
A possible role of the muscle bundles inserting into the medial surface of the condylar process during jaw movement. Schematic illustrations on the anterior (a and b) and superior (c) aspects of the right mandible and sphenoid. Although two units of the lateral pterygoid, indicated by red and blue arrows, are insufficient to contribute to the coordination of the mandibular movements in the frontal and horizontal planes (a), three units of the lateral pterygoid with a unit indicated by the green arrow could contribute to the coordination of the mandibular movements in the multidimensional plane (b and c). Blue arrow, a unit of the lateral pterygoid which originates from the lateral plate of the pterygoid process and inserts into the pterygoid fovea of the condylar process; Green arrow, a unit of the lateral pterygoid which originates from the lateral plate of the pterygoid process and inserted into the medial surface of the condylar process; PpL, lateral plate of the pterygoid process; Red arrow, a unit of the lateral pterygoid originating from the inferior surface of the greater wing. *Med*, medial; *Post*, posterior; and *Sup*, superior

Our findings highlight a few important clinical insights. As described by Posselt (1957), jaw movements are three‐dimensionally limited and coordinated. Some studies have suggested that the lateral pterygoid might act as one muscle and the subunits within the muscle might generate the force for coordinating multidimensional jaw movements (Hannam & McMillan, 1994; Hiraba et al., 2000; Murray, 2012; Widmalm et al., 1987). Conventionally, the insertion of the lateral pterygoid was limited on the pterygoid fovea (Figure 5(a)). However, since the muscle bundle inserting into the medial surface of the condylar process had a broad insertion and distinct origin, it can act as one of the subunits of the lateral pterygoid for coordinating the jaw movements on the multidimensional planes (Figure 5(b)). In addition, some studies on the electromyography already demonstrated that superomedial zone of the lower head of the lateral pterygoid and medial zone of its upper head appeared to be important in initiating contralateral and protrusive jaw movements (Murray et al., 2007; Phanachet et al., 2001, 2003). If these medial zones of the upper and lower heads of the lateral pterygoid inserted into the medial surface of the condylar process, its different insertion dimension from the pterygoid fovea on the horizontal plane may enable the first activation of the medial zone of the lateral pterygoid in contralateral and protrusive jaw movements (Figure 5(c)).

This study had some limitations. Firstly, it was a purely anatomical investigation and was limited to cadaveric specimens. Therefore, we could not prove the mechanism of the coordination of the jaw movements. Moreover, we could not exclude the possibility that the advanced age of the donors affected our findings. Additional biomechanical studies or studies involving clinical case imaging are needed to validate our findings.

In conclusion, the muscle bundle of the lateral pterygoid that inserted into the medial surface of the condylar process had a broad insertion area occupying approximately one‐third of the total insertion and had a distinct origin on the posterior region of the lateral plate of the pterygoid process. As one of the subunits within the lateral pterygoid, this muscle bundle can play an essential role in coordinating multidimensional jaw movements.

## CONFLICT OF INTEREST

The authors declare no conflicts of interest.

## AUTHOR CONTRIBUTIONS

Sasin Sritara contributed to the conception, design, acquisition of data, and critical revision of the manuscript. Masahiro Tsutsumi contributed to the conception, design, acquisition of data, data analysis and interpretation, and drafting of the manuscript. Keiko Fukino contributed to the conception, design, data interpretation, and critical revision of the manuscript. Yoshiro Matsumoto contributed to the conception and critical revision of the manuscript. Takashi Ono contributed to the conception, supervision of the work, and critical revision of the manuscript. Keiichi Akita contributed to the conception, design, data interpretation, supervising the work, critical revision of the manuscript, commenting on drafts, and the final version of the manuscript. All authors gave their final approval and agree to be accountable for all aspects of the work.

## Data Availability

Data sharing is not applicable to this article as no new data were created or analyzed in this study.
